# Supplementary material for: Intensive Motion Style Acupuncture Treatment (MSAT) Is Effective for Patients with Acute Whiplash Injury: A Randomized Controlled Trial
Source: J Clin Med. 2020 Jul 2;9(7):2079. doi: 10.3390/jcm9072079 (PMC7408694; doi:10.3390/jcm9072079)
Supplement: Supplementary file 1 [file jcm-09-02079-s001.pdf]

Supplementary Materials

Table 1. Results of a pilot study comparing between MSAT and control groups.

	Outcome change[1]		Mean difference[2]	Pooled SD	Effect size
	MSAT (N=10)	Control (N=9)			
**NRS score for neck pain**	-2.00±1.41	-0.56±1.24	1.44 (0.15, 2.74)	1.32	1.09
**NRS score for arm pain**	-1.30±1.89	-0.67±1.41	0.63 (-1.00, 2.26)	1.89	0.34
**ROM for flexion**	15.00±6.24	4.44±7.68	-10.56 (-17.30, -3.81)	7.03	1.50
**ROM for extension**	23.00±5.87	6.11±9.28	-16.89 (-24.32, -9.46)	7.85	2.15
**ROM for right flexion**	13.00±13.17	3.89±8.58	-9.11 (-20.01, 1.79)	10.97	0.83
**ROM for left flexion**	18.00±8.56	5.00±9.68	-13.00 (-21.83, -4.17)	9.17	1.42
**ROM for right rotation**	26.50±11.56	10.00±13.46	-16.50 (-28.61, -4.39)	12.60	1.31
**ROM for left rotation**	24.50±12.57	8.33±17.32	-16.17 (-30.70, -1.63)	15.27	1.09

Notes: In the pilot study, outcomes were measured at baseline and after each MSAT session, with no follow-up period. Twenty participants were included (MSAT: 10, control: 10). One patient in the control group withdrew. Complete case analysis was performed. [1]Difference between the baseline value and the value after three sessions of MSAT; values are reported as mean ± standard deviation. [2]Mean difference in the outcome change between the two groups; values are reported with 95% confidence intervals. **Abbreviations:** MSAT, motion style acupuncture treatment; NRS, numeric rating scale; ROM, range of motion.

Table 2. Chuna manual therapy techniques used in this study.

**Chuna techniques for the cervical spine**	
Distraction	Supine position cervical distraction method
	Prone position cervical distraction method
Correction	Supine position cervical correction method
	Supine position cervical JS distraction and correction method
	Supine atlas correction method
	Supine occipital correction method

Table 3. Details of motion style acupuncture treatment (MSAT) and traditional acupuncture treatment based on the STRICTA 2010 checklist.

Item	Details
**1. Acupuncture rationale**	**1a) Style of acupuncture:**<br>MSAT: 3 days of motion style acupuncture + Korean traditional acupuncture<br>Control: Korean traditional acupuncture
	**1b) Reasoning for treatment provided, based on historical context, literature sources, and/or consensus methods, with references where appropriate:**<br>MSAT: Used by Korean medicine doctors [1,2] and based on the consensus of a group of clinical experts<br>Korean traditional: Based on the textbook of acupuncture and commonly used by Korean medicine doctors
	**1c) Extent to which the treatment was varied:**

	MSAT: Each treatment is performed for about 10 minutes, but the degree and frequency of movement can be adjusted at the discretion of the doctor.<br>Korean traditional: 6-12 needles among essential and optional acupoints
**2. Details of needling**	**2a) Number of needle insertions per subject per session:**<br>MSAT: 6 acupoints/Korean traditional: 6-12 acupoints
	**2b) Names (or location if no standard name) of points used (unilateral/bilateral):**<br>MSAT: both sides of the upper trapezius.<br>Korean traditional: essential - SI15, TE15, LI16 bilateral<br>optional - GB20, BL10, SI14 bilateral, GV14 unilateral
	**2c) Depth of insertion, based on a specified unit of measurement or a particular tissue level:**<br>MSAT: 5-10 mm based on the depth of upper trapezius<br>Korean traditional: based on the physician's judgment
	**2d) Response sought (e.g., *de qi* or muscle twitch response):**<br>MSAT: None/Korean traditional: *de qi*
	**2e) Needle stimulation (e.g., manual, electrical):**<br>MSAT: manual stimulation during movement<br>Korean traditional: electrical stimulation
	**2f) Needle retention time:**<br>MSAT: about 10 minutes/Korean traditional: 15 minutes
	**2g) Needle type (diameter, length, and manufacturer or material):**<br>0.25 * 30 mm, stainless steel, Dong-bang medical, Korea
**3. Treatment regimen**	**3a) Number of treatment sessions:**<br>MSAT: 3 sessions/Korean traditional: 8-26 sessions
	**3b) Frequency and duration of treatment sessions:**<br>MSAT: once a day for 3 days/Korean traditional: twice a day for 5-13 days
**4. Other components of treatment**	**4a) Details of other interventions administered to the acupuncture group (eg, moxibustion, cupping, herbs, exercises, and lifestyle advice):**<br>Integrative Korean medicine treatment such as acupuncture, phamacopuncture, chuna manual therapy, herbal medicine, etc.
	**4b) Setting and context of treatment, including instructions to practitioners and information and explanations to patients:**<br>None in particular
**5. Practitioner background**	**5) Description of participating acupuncturists (qualification or professional affiliation, years in acupuncture practice, other relevant experience):**<br>Korean medicine doctor with at least 3 years of clinical experience and affiliated to Bucheon Jaseng hospital.
**6. Control or comparator interventions**	**6a) Rationale for the control or comparator in the context of the research question, with sources that justify this choice:**<br>For the purpose of the study evaluating the effectiveness of additional treatment with MSAT in traditional Korean medicine treatment, the control was defined as a group that received integrative Korean medicine treatment only.

	**6b) Precise description of the control or comparator. If sham acupuncture or any other type of acupuncture-like control is used, provide details as for items 1 to 3 above:**<br>Integrative Korean medicine treatment such as acupuncture, phamacopuncture, chuna manual therapy,<br>herbal medicine, etc. Details related to acupuncture are mentioned for items 1-3.

MSAT, motion style acupuncture treatment

Table 4. Study schedule and measurements at each visit.

	**Study period**										
	**Enrolment**	**Allocation**	**Active treatment**							Follow-up	
**Days[1]**	**1**	**1**	**2[2]**	**2[3]**	3	4	5	8	d/c	14	90
**Enrolment**											
Eligibility screen	○										
Informed consent	○										
Credibility and expectancy	○										
Medical history	○										
Allocation		○									
**Interventions**											
MSAT+IKM			○		○	○					
IKM			○		○	○	○	○			
**Assessment**											
Check symptoms and Medicine change		○	○		○	○	○	○	○	○	○
NRS score for neck pain[4]	○	○	○	○	○	○	○	○	○	○	○
VAS score for neck pain[4]		○	○	○	○	○	○	○	○		
Cervical ROM[4]		○	○	○	○	○	○	○	○		
NRS score for arm pain[4]		○	○	○	○	○	○	○	○	○	○
VAS score for arm pain[4]		○	○	○	○	○	○	○	○		
NDI		○					○	○	○	○	○
EQ-5D		○					○	○	○	○	○
PGIC							○	○	○	○	○
Adverse events		○	○		○	○	○	○	○	○	○

Notes: [1]Days from admission [2]Outcomes before treatment on day 2 [3]Outcomes after treatment on day 2 [4]NRS scores for neck and arm pain, VAS scores for neck and arm pain, and ROM of the neck were additionally measured after treatment on the second day. **Abbreviations:** d/c, discharge; MSAT, motion style acupuncture treatment; IKM, integrative Korean medicine treatment; NRS, numeric rating scale; VAS, visual analog scale; ROM, range of motion; NDI, neck disability index; EQ-5D, EuroQol-5 dimension; PGIC, patient global impression of change.

Table 5. Cases of adverse events during study period by treatment group.

		**MSAT (N=48)**	**Control (N=49)**
**Total cases**		13	7
**Severity[1]**	**Mild**	12	6
	**Moderate**	1	0
	**Severe**	0	0
**Gastro Intestinal symptoms**			
- **Diarrhea**		8	4
- **Nausea, Heartburn**		2	1
**Skin related symptoms (rash, itchy, vesicle)**		2	2
**Dizziness**		1	0

[1] Severity was classified according to Spilker, et al. [3].Mild, did not impair the participant's normal activities of daily living [ADLs], caused minimal discomfort, and required no additional treatment; moderate, significantly impaired the participant's normal ADLs and may have required treatment, but they resolved after treatment; severe, severely impaired the participant's normal ADLs, required intense treatment, and left sequelae.

1. Shin, J.; Boone, W.; Kim, P.; So, C. Acute and long term benefits of motion style treatment (MST): two case reports. *International Journal of Clinical Acupuncture* **2007**, *16*, 85.
2. Shin, J.-S.; Ha, I.-H.; Lee, J.; Choi, Y.; Kim, M.-r.; Park, B.-Y.; Shin, B.-C.; Lee, M.S. Effects of motion style acupuncture treatment in acute low back pain patients with severe disability: a multicenter, randomized, controlled, comparative effectiveness trial. *PAIN®* **2013**, *154*, 1030-1037.
3. Song, K.-J.; Choi, B.-W.; Kim, S.-J.; Yoon, S.-J. Cross-cultural adaptation and validation of the Korean version of the neck disability index. *Journal of the Korean Orthopaedic Association* **2009**, *44*, 350-359.

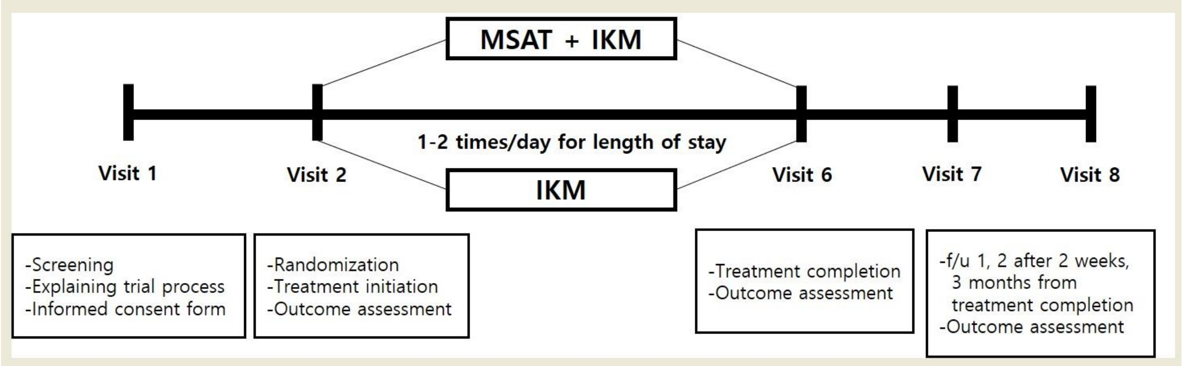

Figure 1. Research process of the study.

© 2020 by the authors. Licensee MDPI, Basel, Switzerland. This article is an open access article distributed under the terms and conditions of the Creative Commons Attribution (CC BY) license (http://creativecommons.org/licenses/by/4.0/).
